# Family income and health in Canada: a longitudinal study of stability and change

**DOI:** 10.1186/s12889-021-10397-5

**Published:** 2021-02-10

**Authors:** Adam Vanzella-Yang, Gerry Veenstra

**Affiliations:** Department of Sociology, University of British Columbia, Vancouver, British Columbia Canada

**Keywords:** Self-rated health, Longstanding illness or health problem, Family income, Stability, Volatility, Trajectory, Canada

## Abstract

**Background:**

Extensive research has shown strong associations between income and health. However, the health effects of income dynamics over time are less known. We investigated how stability, volatility and trajectory in family incomes from 2002 to 2011 predicted (1) fair/poor self-rated health and (2) the presence of a longstanding illness or health problem in 2012.

**Methods:**

The data came from the 2012 wave of the Longitudinal and International Study of Adults linked to annual family income data for 2002 to 2011 from the Canada Revenue Agency. We executed a series of binary logistic regressions to examine associations between health and average family income over the decade (Model 1), number of years in the bottom quartile (Model 2) and top quartile (Model 3) of family incomes, standard deviation of family incomes (Model 4), absolute difference between family income at the end and start of the period (Model 5), and number of years in which inflation-adjusted family income went down by more than 1% (Model 6) and up by more than 1% (Model 7) from 1 year to the next. The analyses were conducted separately for women and men.

**Results:**

Average family income over the decade was strongly associated with both self-rated health and the presence of a longstanding illness or health problem. More years spent in the bottom quartile of family incomes corresponded to elevated odds of fair/poor self-rated health and the presence of a longstanding illness or health problem. Steady decreases in family income over the decade corresponded to elevated odds of fair/poor self-rated health for men and more years spent in the top quartile of family incomes over the decade corresponded to elevated odds of fair/poor self-rated health for women.

**Conclusion:**

Previous studies of the association between family income and health in Canada may have overlooked important issues pertaining to family income stability and change that are impactful for health.

**Supplementary Information:**

The online version contains supplementary material available at 10.1186/s12889-021-10397-5.

## Background

Previous research has established the existence of strong associations between family or household income and health in Canada (e.g. [1–6]). Most of these studies are cross-sectional and therefore incapable of addressing health-related issues pertaining to income dynamics over time – a topic of growing research and public health interest [7–11]. In this study we investigated how stability, volatility and trajectory in incomes over a decade predicted two health outcomes: fair/poor self-rated health and the presence of a longstanding illness or health problem.

Family incomes can change from year to year with the formation or dissolution of marital or common-law partnerships, adult children leaving home to form their own families, changes in occupations, periods of unemployment, onetime financial windfalls or losses, and so forth. The volatility of family incomes means that a measure of family income in a given year can misrepresent the stable nature of people’s economic circumstances over longer periods of time [10]. Consistent with this interpretation, Benzeval and Judge [12] found that average income over a five-year period was a stronger predictor of self-rated health in the following year than income in any specific year. Benzeval and Judge also found that people who experienced persistent poverty over the 5 years had the worst self-rated health and were the most likely to report a limiting illness [12]. Similarly, McDonough and colleagues [13] found that persistent low income over a five-year period corresponded to elevated odds of mortality, and McDonough and Berglund found that histories of poverty affected health status [14]. More recently, a Danish study found that persistently low family income across two age periods (45–49 and 55–59 years of age) was associated with greater risk of hospitalization and mortality in later life, with stronger effects among men [7]. Thus, we test whether persistent income disadvantage or advantage is associated with health while controlling for average income.

*H1: The greater the number of years in the bottom quartile the greater the likelihood of poor health, controlling for average income.*

*H2: The greater the number of years in the top quartile the lower the likelihood of poor health, controlling for average income.*

It is also possible that income-related volatility itself has a deleterious effect on health. A recent study of young adults in the United States found that income volatility was associated with greater risk of cardiovascular disease and all-cause mortality net of demographic, lifestyle, and physical and mental health controls [9]. Income volatility may also result in uncertainty regarding the future, poor health behaviors and greater risk of acute and chronic health care outcomes [8]. Prause and colleagues [15], however, found that a higher variance of incomes in fact corresponded to lower depression scores in the United States. Similarly, Benzeval and Judge [12] found that the standard deviation of incomes was positively associated with self-rated health, although this measure of volatility was also positively correlated with income change which might explain this finding. In South Korea, income volatility was positively associated with higher risk of depression among elderly people living alone and lower risk of depression among elderly people living with children [16]. Our third hypothesis therefore pertains to the health effects of income volatility over a decade.

*H3: The higher the standard deviation of incomes the greater the likelihood of poor health, controlling for average income.*

Dramatic changes in income can also affect health. Benzeval and Judge [12], comparing the monetary difference at the start and end of a six-year period, found that income differences corresponded to better self-rated health and lower risks of a limiting illness. McDonough and colleagues [13] found that one or more income losses of over 50% during a five-year period corresponded to greater odds of mortality. Similarly, Elfassy and colleagues [9] found that the number of income drops of over 25% from 1 year to another was associated with greater risk of cardiovascular disease and all-cause mortality among young adults. This previous research informs our fourth hypothesis:

*H4: The greater the income difference between the end and the start of the decade the greater the likelihood of poor health, controlling for income at the start of the decade.*

Short-term effects of decreasing incomes might include frustration and stress while long-term effects of steadily decreasing incomes might include material shortages and reduced purchasing power, feelings of deprivation and diminished social ties [10]. Research from Sweden found that a steady downward trend in income was associated with greater odds of poor self-rated health for men [10]. A Danish study likewise found that a downward family income trajectory in midlife increased the risk of mortality and hospitalization in later life, with stronger effects among men [7]. Similar results were obtained for upward trajectory of family income, though with weaker effects [7]. Thus the last two hypotheses of our study:

*H5: Steadily increasing incomes are associated with lower likelihood of poor health, controlling for average income.*

*H6: Steadily decreasing incomes are associated with greater likelihood of poor health, controlling for average income.*

This paper addresses a range of issues pertaining to the health effects of income dynamics over time in the understudied Canadian context, thereby contributing to a better understanding of the well-known links between socioeconomic status and health.

## Methods

We used data from the Longitudinal and International Study of Adults (LISA) collected by Statistics Canada in 2012 (wave one). Detailed descriptions of the LISA can be found in the Statistics Canada webpage [17] and in previous publications by the authors of this paper [18, 19]. In the present study, respondents were linked to their T1 Family File income tax data for each year from 2002 to 2011. We restricted our analyses to survey respondents who were 25 years of age or older in 2002, reducing the sample to approximately 16,200 respondents. We also restricted our analyses to respondents who had valid family income data for 2002, further reducing the sample to approximately 14,500 respondents. A comparison of the study participants with valid income data for 2002 to the participants dropped at this stage indicated that the dropouts were relatively likely to have immigrated to Canada, to be young, single and highly educated and to have highly educated parents. The other variables used in our study had small amounts of missing data. Accordingly, we applied listwise deletion to produce a final sample comprised of approximately 13,000 people (6000 men and 7000 women). Statistics Canada’s confidentiality policies when using LISA data linked to Canada Revenue Agency (CRA) income data require sample sizes to be rounded and prevent us from providing descriptive statistics of the variables used in our study. Descriptive statistics for some of the variables were provided for reviewer use only.

The dependent variables were self-rated health and the presence of a longstanding illness or health problem, assessed in 2012. Respondents were asked ‘In general, would you say your health is excellent, very good, good, fair or poor?’ We dichotomized this variable to distinguish between fair/poor and excellent/very good/good health. Respondents were also asked ‘Do you have any longstanding illnesses or longstanding health problems that have lasted or are expected to last for 6 months or more?’ which was coded yes or no.

The independent variables pertained to Census family income. A Census family is comprised of a married couple with or without children of either or both spouses, a common-law couple with or without children of either or both partners, a lone parent living with at least one child or a person living alone. We procured annual family incomes from 2002 to 2011 which we adjusted for inflation, equivalized by dividing by the square root of family size and logged to address right skewness. We calculated average annual family income for the period 2002 to 2011 to assess stability in incomes and the standard deviation of the logged, inflation-adjusted equivalized family incomes from 2002 to 2011 to assess volatility in incomes. We then standardized all of these variables to facilitate their interpretation in logistic regression models. Further to the issue of income stability, we calculated the number of years a respondent’s family income was in the bottom quartile of incomes as well as the number of years a respondent’s family income was in the top quartile of incomes. To assess changes in incomes we calculated the difference between logged and inflation adjusted (but not standardized) family incomes in 2011 and 2002. We also calculated a downward trajectory variable that was initially set to zero and then increased by one for every year from 2002 to 2011 in which logged, inflation adjusted (but not standardized) family income was more than 1% lower than it was in the previous year. Lastly, we calculated an upward trajectory variable that was initially set to zero and then increased by one for every year from 2002 to 2011 in which logged, inflation adjusted (but not standardized) family income was more than 1% higher than it was in the previous year. We used 1% instead of the higher thresholds used in other studies [10, 12, 13, 15] because very few of our respondents had any year-to-year comparisons where their family income rose or fell by more than 2%.

The control variables were age in years, immigrant status and parental education as assessed in 2012. We also control for marital status at the start and end of the decade (2002 and 2011), and we indirectly control for family size by equivalizing family incomes using the OECD method, i.e. dividing it by the square root of family size [20]. We did not control for variables such as education and labour market status that may have changed between 2002 and 2012 as these variables were not available in the family income files over the decade. Immigrant status distinguished between respondents born in Canada and immigrants to Canada. Respondents recorded the highest educational attainment of their mother or female guardian and their father or male guardian. From these we created a single variable assessing highest parental education that distinguished between less than high school, high school diploma or equivalent, certificate or diploma from a technical school, community college or university, and bachelor degree or higher.

We ran a series of binary logistic regressions on each health variable separately for women and men as summarized in Tables 1 and 2. The tables contain odds ratios and predicted probabilities generated from these models as well as risk ratios and risk differences calculated from the predicted probabilities [21]. Model 1 describes the association between average family income and health over the decade. Models 2 and 3 describe associations between health and the number of years in the bottom quartile and top quartile, respectively, controlling for average income. Model 4 describes the association between income volatility and health, controlling for average income. Model 5 describes the association between health and income differences at the end and start of the decade, controlling for income at the start of the decade. Finally, Models 6 and 7 show associations between health and downward and upward income trajectories, respectively, controlling for average income. Results for models describing associations between yearly family income indicators and the two health outcomes are provided in supplementary Tables 1 and 2 (we advise readers to interpret these supplementary results as descriptive). Statistics Canada provided person weight and 1000 bootstrap weights which we applied in our analyses to produce more accurate point estimates and standard errors, respectively. Statistical analyses were executed using Stata 16. Approval for the study was granted by the Behavioural Research Board at The University of British Columbia.

**Table 1:** Regressing family income variables on fair/poor self-rated health in 2012

	***Women***					***Men***				
	*OR (95% CI)*	*PP1*	*PP2*	*RR*	*RD*	*OR (95% CI)*	*PP1*	*PP2*	*RR*	*RD*
*Model 1*										
Average income	0.47 (0.39–0.57)	0.17	0.09	0.53	0.08	0.54 (0.44–0.65)	0.18	0.11	0.60	0.07
*Model 2*										
Years in bottom $ quartile	1.10 (1.06–1.14)	0.11	0.12	1.08	0.01	1.08 (1.04–1.12)	0.13	0.13	1.07	0.01
Average income	0.72 (0.59–0.87)					0.72 (0.58–0.89)				
*Model 3*										
Years in top $ quartile	1.12 (1.05–1.18)	0.14	0.15	1.09	0.01	1.05 (0.99–1.13)	0.15	0.15	1.04	0.01
Average income	0.33 (0.24–0.45)					0.45 (0.31–0.64)				
*Model 4*										
SD of incomes	0.96 (0.83–1.10)	0.15	0.15	0.97	0.00	1.16 (0.98–1.37)	0.15	0.17	1.12	0.02
Average income	0.48 (0.40–0.59)					0.49 (0.40–0.61)				
*Model 5*										
2011 $ – 2002 $	0.65 (0.53–0.81)	0.17	0.12	0.71	0.05	0.63 (0.52–0.76)	0.18	0.12	0.69	0.06
2002 income	0.51 (0.43–0.61)					0.56 (0.46–0.68)				
*Model 6*										
Downward trajectory of $	0.99 (0.64–1.16)	0.15	0.15	0.99	0.00	1.50 (1.03–2.21)	0.15	0.21	1.36	0.05
Average income	0.47 (0.39–0.57)					0.51 (0.42–0.62)				
*Model 7*										
Upward trajectory of $	1.29 (0.92–1.80)	0.15	0.18	1.21	0.03	1.36 (0.86–2.15)	0.15	0.19	1.26	0.04
Average income	0.46 (0.38–0.56)					0.52 (0.43–0.64)				

Note: For average income, SD of incomes and 2011 $ – 2002 $, PP1and PP2 are the predicted probabilities at 0.5 standard deviations below and above their means, respectively. For years in bottom quartile, years in top quartile, downward trajectory of $ and upward trajectory of $, PP1and PP2 are the predicted probabilities at 0 and 1, respectively. RR (risk ratio) is PP2 divided by PP1 and RD (risk difference) is the absolute value of PP1 subtract PP2. Each model controls for age in years, immigrant status, marital status and parental education. Person and replicate weights are applied to each model

**Table 2:** Regressing family income variables on the presence of a longstanding illness or health problem in 2012

	***Women***					***Men***				
	*OR (95% CI)*	*PP1*	*PP2*	*RR*	*RD*	*OR (95% CI)*	*PP1*	*PP2*	*RR*	*RD*
*Model 1*										
Average income	0.79 (0.72–0.86)	0.43	0.37	0.87	0.06	0.87 (0.79–0.97)	0.40	0.37	0.92	0.03
*Model 2*										
Years in bottom $ quartile	1.04 (1.02–1.07)	0.38	0.39	1.02	0.01	1.05 (1.02–1.08)	0.35	0.36	1.03	0.01
Average income	0.86 (0.78–0.95)					0.95 (0.86–1.06)				
*Model 3*										
Years in top $ quartile	1.02 (0.98–1.06)	0.40	0.41	1.01	0.00	0.99 (0.95–1.03)	0.39	0.38	0.99	0.00
Average income	0.74 (0.62–0.90)					0.90 (0.75–1.08)				
*Model 4*										
SD of incomes	0.97 (0.88–1.05)	0.41	0.41	0.98	0.01	1.04 (0.94–1.14)	0.38	0.39	1.02	0.01
Average income	0.80 (0.72–0.89)					0.85 (0.75–0.97)				
*Model 5*										
2011 $ – 2002 $	0.81 (0.74–0.88)	0.43	0.38	0.89	0.05	0.88 (0.78–1.00)	0.39	0.37	0.93	0.03
2002 income	0.77 (0.71–0.84)					0.87 (0.78–1.74)				
*Model 6*										
Downward trajectory of $	1.21 (0.98–1.48)	0.41	0.45	1.11	0.04	1.05 (0.82–1.34)	0.38	0.39	1.03	0.01
Average income	0.76 (0.69–0.84)					0.87 (0.77–0.97)				
*Model 7*										
Upward trajectory of $	1.04 (0.83–1.30)	0.41	0.42	1.02	0.01	1.04 (0.81–1.33)	0.38	0.39	1.02	0.01
Average income	0.78 (0.71–0.86)					0.87 (0.77–0.97)				

Note: For average income, SD of incomes and 2011 $ – 2002 $, PP1and PP2 are the predicted probabilities at 0.5 standard deviations below and above their means, respectively. For years in bottom quartile, years in top quartile, downward trajectory of $ and upward trajectory of $, PP1and PP2 are the predicted probabilities at 0 and 1, respectively. RR (risk ratio) is PP2 divided by PP1 and RD (risk difference) is the absolute value of PP1 subtract PP2. Each model controls for age in years, immigrant status, marital status and parental education. Person and replicate weights are applied to each model

## Results

Tables 1 and 2 summarize the results of modelling average family income (Model 1) and time spent in the bottom quartile (Model 2) and top quartiles (Model 3) of family incomes on the two health indicators. Average income was more strongly associated with fair/poor self-rated health (OR = 0.47 with 95% CI = 0.39–0.57 for women; OR = 0.54 with 95% CI = 0.44–0.65 for men) than with the presence of a longstanding illness or health problem (OR = 0.79 with 95% CI = 0.72–0.86 for women; OR = 0.87 with 95% CI = 0.79–0.97 for men), and associations appeared to be stronger among women than among men. As hypothesized, being stably poor over the decade corresponded to elevated risks of fair/poor self-rated health (OR = 1.10 with 95% CI = 1.06–1.14 for women; OR = 1.08 with 95% CI = 1.04–1.12 for men) and the presence of a longstanding illness or health problem (OR = 1.04 with 95% CI = 1.02–1.07 for women; OR = 1.05 with 95% CI = 1.02–1.08 for men), controlling for average family income. Remarkably, more years spent in the top quartile of family incomes over the decade also corresponded to elevated risks of fair/poor self-rated health for women (OR = 1.12 with 95% CI = 1.05–1.18), controlling for average family income.

Model 4 in Tables 1 and 2 summarize the results of modelling the standard deviation of family incomes on the two health indicators while controlling for average family income. Volatility was not meaningfully associated with either health outcome for women nor men, counter to our hypothesis.

Model 5 in Tables 1 and 2 indicate that increases in family income over the decade corresponded to lower risks of fair/poor self-rated health (OR = 0.65 with 95% CI = 0.53–0.81 for women; OR = 0.63 with 95% CI = 0.52–0.76 for men) and the presence of a longstanding illness or health problem (OR = 0.81 with 95% CI = 0.74–0.88 for women; OR = 0.88 with 95% CI = 0.78–1.00 for men), controlling for family income at the start of the decade. Family income in 2002 was also statistically significant in all of the models, however, and was as or more strongly associated with each health indicator than was income change.

Tables 1 and 2 also include the results of modelling the trajectory of family incomes on health. Model 6 shows that a steady downward trajectory of family income over the decade corresponded to a higher risk of fair/poor self-rated health for men (OR = 1.50 with 95% CI = 1.03–2.21 as hypothesized, but was of little consequence for the self-rated health of women. Neither kind of steady trajectory was meaningfully associated with the presence of a longstanding illness or health problem (Models 6 and 7).

## Discussion

Average family income was significantly associated with both health indicators. These associations were stronger for fair/poor self-rated health and for women. However, we suspect that these associations are not causal in nature. Recent research using fixed effects models to describe associations between changes in family income and changes in health found no evidence of strong causal effects of income on health over a short period of time [18, 22–24]. In contrast, research from the United States has shown significant effects of long-term (more than 10 years) but not short-term (2 years) income exposures on self-rated health [25] As new waves of the LISA become available in the future, a more comprehensive version of the present study should be undertaken, accounting for initial health status and other possible confounders which we could not address using data from the linked historical family files from the CRA.

It is only when we consider stable presence in the bottom or top income quartiles that we uncover associations that are potentially causal. Here we find that more years spent in the bottom quartile of family incomes over the decade corresponded to elevated risks of fair/poor self-rated health and the presence of a longstanding illness or health problem, controlling for average income. This is consistent with previous research showing the importance of relative comparison in addition to absolute material circumstances in the production of health outcomes [26]. Alternatively, these results could reflect reverse causality whereby poor health at the start of the decade leads to being stably poor over the decade. Consistent with the latter interpretation, Salm [27] found that poor health influenced job loss but job loss had no effect on health while Stewart [28] found that people with poor health experienced longer spells of unemployment.

We were intrigued to discover that more years spent in the top quartile of family incomes over the decade was also associated with elevated risks of fair/poor self-rated health for women. Though directionally contrary to our hypothesis, this finding may reflect a societal context where gender essentialisms persist despite structural and material changes pushing toward equality [29]. Presence in the upper tiers of income for women often means having to succeed in traditionally male-typed professional or managerial jobs [30]. Breaking these cultural barriers while also dealing with a still uneven distribution of housework [31] and gendered expectations about family responsibilities [32] may increase exposure to stressors and therefore be the cause of poorer health among women who spent more years in top family income quartile. It is worth pointing out, however, that these associations were almost statistically significant for men as well, which could point to employment related stressors pertaining to high earning jobs in general.

Stably low or high incomes were associated with relatively poor health but so too were changes in income from 2002 to 2011. We found that the greater the difference in family incomes when comparing the end and the start of the decade the lower the odds of poor health, controlling for family income at the start of the decade. We also found that steadily decreasing family incomes corresponded to elevated risks of fair/poor self-rated health, controlling for average family income, but only for men. In light of research which suggests that lifestyle factors mediate the association between income and health [33], declining incomes could entail a decreased ability to engage in conscientious eating and exercising. Another explanation would be that declining incomes signify a departure from hegemonic ideals of masculinity that are entwined with upper-class economic status, which may in turn cause a decline in health and well-being [34]. Alternatively, the reverse causality explanation could again be at play wherein poor health leads to steadily declining incomes over time. The lack of an association between declining incomes and health for women might reflect the fact that women may have more social support to mitigate these negative effects [35].

### Strengths and limitations

One of the biggest strengths of our study is the use of unprecedented valid and precise measures of current and historical family income, indicators that typically have sizeable amounts of missing data and are much less valid and precise when self-reported in surveys. The majority of previous studies have used self-reported income which often has sizeable amounts of missing data that may be missing not at random (MNAR) instead of missing at random (MAR) or missing completely at random (MCAR). MNAR data compromise the validity of indicators of family or household income and the representativeness of studies that utilize them. Another notable strength of the analysis pertains to the relatively lengthy period of time covered (2002–2012) compared to previous studies that were limited to substantially shorter periods of time [10, 12, 13, 15].

However, the unique nature of our dataset, cross-sectional survey data linked to 10 years of historical income data, comes with limitations. We were unable to address the issue of health selection by controlling for initial health status or only including respondents who were healthy in 2002. Additionally, we were able to control for some (e.g., age, immigrant status, parental education, marital status and family size) but not all of the factors that could produce spurious associations between family income and health (e.g., genetics, personality, parental wealth, health in childhood, education, labour market status). This means that reverse causal directionality and causal confounding are strong contenders for explaining the empirical associations reported here. Lastly, respondents had to have valid income data for 2002 to be included in the analyses which eliminated recent immigrants and younger people from the working sample. This means that our study is not fully representative of the Canadian population writ large.

## Conclusion

Our results show that issues pertaining to family income stability and change may be impactful for health and should be further investigated. In particular, our findings suggest possible gender differences in how income dynamics over time shape health. While most findings were similar for women and men, we found that a downward trajectory of family incomes over the decade corresponded to elevated odds of fair/poor self-rated health among men and that more years spent in the top quartile of family incomes over the decade corresponded to elevated odds of fair/poor self-rated health among women. As future waves of the LISA become available, a more fulsome longitudinal analysis of the issues investigated in this study may become possible. The availability of future survey waves would allow researchers to address the issue of health selection and include important control variables such as education and employment status, which were not available in the family files for our investigation, and accordingly obtain a more accurate representation of how income stability and change over time may affect health.

## Supplementary Information

**Additional file 1: Supplementary Table S1.** Year-stratified analysis of family income on fair/poor self-rated health in 2012. **Supplementary Table S2.** Year-stratified analysis of family income on the presence of a longstanding illness or health problem in 2012

## Data Availability

Data cannot be shared publicly because of Statistics Canada’s confidentiality policies when using survey data linked to Canada Revenue Agency income data. Data are available from the Research Data Centres in 32 universities across Canada for researchers who meet the criteria for access to confidential data (https://www.statcan.gc.ca/eng/microdata/data-centres). The analyses for this study were conducted in the Research Data Centre at UBC-Vancouver.

## Abbreviations

CRA: Canada Revenue Agency
LISA: Longitudinal and International Study of Adults
MAR: Missing at random
MCAR: Missing completely at random
MNAR: Missing not at random

## References

[1] Cairney J. Socio-economic Status and Self-Rated Health Among Older Canadians. Can J Aging / La Rev Can du Vieil [Internet]. 2010/11/29. 2000;19(4):456–78. Available from: https://www.cambridge.org/core/article/socioeconomic-status-and-selfrated-health-among-older-canadians/2E9554ECDC039288F3E6298E4CBE2E38

[2] Dunn JR, Veenstra G, Ross N (2006). Psychosocial and neo-material dimensions of SES and health revisited: predictors of self-rated health in a Canadian national survey. Soc Sci Med [Internet]..

[3] Humphries KH, van Doorslaer E (2000). Income-related health inequality in Canada. Soc Sci Med [Internet]..

[4] McLeod CB, Lavis JN, Mustard CA, Stoddart GL (2003). Income Inequality, Household Income, and Health Status in Canada: A Prospective Cohort Study. Am J Public Health [Internet].

[5] Orpana HM, Lemyre L, Kelly S (2007). Do stressors explain the association between income and declines in self-rated health? A longitudinal analysis of the national population health survey. Int J Behav med [Internet].

[6] Prus SG (2011). Comparing social determinants of self-rated health across the United States and Canada. Soc Sci Med [Internet]..

[7] Ahrenfeldt LJ, Pedersen JK, Thinggaard M, Christensen K, Lindahl-Jacobsen R (2019). Sex differences in health and mortality by income and income changes. J Epidemiol Community Health.

[8] Basu S (2017). Income volatility: A preventable Public Health threat. Am J Public Health.

[9] Elfassy T, Swift SL, Glymour MM, Calonico S, Jacobs DR, Mayeda ER (2019). Associations of income volatility with incident cardiovascular disease and all-cause mortality in a US Cohort: 1990 to 2015. Circulation..

[10] Miething A, Åberg YM (2014). Stability and variability in income position over time: exploring their role in self-rated health in Swedish survey data. BMC Public Health [Internet].

[11] Schöllgen I, Kersten N, Rose U (2019). Income trajectories and subjective well-being: Linking administrative records and survey data. Int J Environ Res Public Health.

[12] Benzeval M, Judge K (2001). Income and health: the time dimension. Soc Sci Med [Internet]..

[13] McDonough P, Duncan GJ, Williams D, House J (1997). Income dynamics and adult mortality in the United States, 1972 through 1989. Am J Public Health.

[14] McDonough P, Berglund P (2003). Histories of poverty and self-rated Health trajectories. J Health Soc Behav [Internet].

[15] Prause J, Dooley D, Huh J. Income volatility and psychological depression. Am J community Psychol [Internet]. 2009;43(1):57–70. Available from: 10.1007/s10464-008-9219-3. 10.1007/s10464-008-9219-3 19130213

[16] Kim S, Subramanian SV (2019). Income volatility and depressive symptoms among Elderly Koreans. Int J Environ Res Public Health.

[17] Statistics Canada [Internet]. Longitudinal and International Study of Adults (LISA). 2011. Available from: https://www23.statcan.gc.ca/imdb/p2SV.pl?Function=getSurvey&Id=119195#a1.

[18] Veenstra G, Vanzella-Yang A. Family income and self-rated health in Canada: using fixed effects models to control for unobserved confounders and investigate causal temporality. Soc Sci Med [Internet]. 2020;250(February):112884. Available from: 10.1016/j.socscimed.2020.112884. 10.1016/j.socscimed.2020.112884 32114260

[19] Veenstra G, Vanzella-Yang A (2020). Does household income mediate the association between education and health in Canada? Scand J Public Health.

[20] OECD. Growing Unequal? Income Distribution and Poverty in OECD Countries. OECD Publishing, editor. Paris; 2008.

[21] Muller CJ, MacLehose RF. Estimating predicted probabilities from logistic regression: different methods correspond to different target populations. Int J Epidemiol [Internet]. 2014 ;43(3):962–970. Available from: 10.1093/ije/dyu029. 10.1093/ije/dyu029 PMC4052139 24603316

[22] Imlach Gunasekara F, Carter K, Blakely T (2011). Change in income and change in self-rated health: systematic review of studies using repeated measures to control for confounding bias. Soc Sci Med [Internet]..

[23] Imlach Gunasekara F, Carter KN, Liu I, Richardson K, Blakely T (2012). The relationship between income and health using longitudinal data from New Zealand. J Epidemiol Community Health [Internet].

[24] Reche E, Konig H-H, Hajek A. Income, self-rated health, and morbidity: A systematic review of longitudinal studies. Int J Environ Res Public Health. 2019;16. 10.3390/ijerph16162884 PMC6720187 31409047

[25] Berry B (2007). Does money buy better health? Unpacking the income to health association after midlife. Heal An Interdiscip J Soc Study Heal Illn Med.

[26] Veenstra G (2005). Social status and health: absolute deprivation or relative comparison, or both?. Heal Sociol Rev.

[27] Salm M (2009). Does job loss cause ill health?. Health Econ [Internet].

[28] Stewart JM (2001). The impact of health status on the duration of unemployment spells and the implications for studies of the impact of unemployment on health status. J Health Econ [Internet].

[29] Ridgeway C. Framed by Gender: Oxford University Press; 2011.

[30] England P. The Gender Revolution: Uneven and Stalled, Gend Soc [Internet]. 2010;24(2):149–66. Available from. 10.1177/0891243210361475.

[31] Guppy N, Sakumoto L, Wilkes R (2019). Social Change and the Gendered Division of Household Labor in Canada. Can Rev Sociol Can Sociol [Internet].

[32] Hirsh CE, Treleaven C, Fuller S. Caregivers, Gender, and The Law: An Analysis of Family Responsibility Discrimination Case Outcomes. Gend Soc [Internet]. 2020 Aug 20;0891243220946335. Available from: 10.1177/0891243220946335.

[33] Christensen VT, Carpiano RM (2014). Social class differences in BMI among Danish women: applying Cockerham’s health lifestyles approach and Bourdieu’s theory of lifestyle. Soc Sci Med [Internet]..

[34] Courtenay WH (2000). Constructions of masculinity and their influence on men’s well-being: a theory of gender and health. Soc Sci Med [Internet].

[35] Turner HA (1994). Gender and social support: taking the bad with the good?. Sex Roles.

