# Supplementary material for: Family income and health in Canada: a longitudinal study of stability and change
Source: BMC Public Health. 2021 Feb 10;21:333. doi: 10.1186/s12889-021-10397-5 (PMC7877036; doi:10.1186/s12889-021-10397-5)
Supplement: Supplementary file 1 — Additional file 1: Supplementary Table S1. Year-stratified analysis of family income on fair/poor self-rated health in 2012. Supplementary Table S2. Year-stratified analysis of family income on the presence of a longstanding illness or health problem in 2012 [file 12889_2021_10397_MOESM1_ESM.docx]

| **Supplementary table 1. Year-stratified analysis of family income on fair/poor self-rated health in 2012** | | | | | | | | | | |
| --- | --- | --- | --- | --- | --- | --- | --- | --- | --- | --- |
|  | ***Women*** | | | | | ***Men*** | | | | |
|  | *OR (95% CI)* | *PP1* | *PP2* | *RR* | *RD* | *OR (95% CI)* | *PP1* | *PP2* | *RR* | *RD* |
| *Model 1*  2002 family income | 0.58 (0.50-0.68) | 0.17 | 0.11 | 0.64 | 0.06 | 0.67 (0.57-0.80) | 0.18 | 0.13 | 0.72 | 0.05 |
| *Model 2*  2003 family income | 0.54 (0.46-0.63) | 0.17 | 0.10 | 0.60 | 0.07 | 0.61 (0.50-0.73) | 0.18 | 0.12 | 0.66 | 0.06 |
| *Model 3*  2004 family income | 0.54 (0.45-0.65) | 0.17 | 0.10 | 0.60 | 0.07 | 0.55 (0.45-0.68) | 0.18 | 0.11 | 0.61 | 0.07 |
| *Model 4*  2005 family income | 0.56 (0.47-0.67) | 0.17 | 0.10 | 0.62 | 0.06 | 0.55 (0.45-0.66) | 0.18 | 0.11 | 0.61 | 0.07 |
| *Model 5*  2006 family income | 0.52 (0.44-0.62) | 0.17 | 0.10 | 0.58 | 0.07 | 0.57 (0.47-0.70) | 0.18 | 0.11 | 0.63 | 0.07 |
| *Model 6*  2007 family income | 0.51 (0.42-0.62) | 0.17 | 0.09 | 0.57 | 0.07 | 0.59 (0.49-0.70) | 0.18 | 0.12 | 0.65 | 0.06 |
| *Model 7*  2008 family income | 0.55 (0.45-0.67) | 0.16 | 0.10 | 0.60 | 0.07 | 0.56 (0.46-0.68) | 0.18 | 0.11 | 0.62 | 0.07 |
| *Model 8*  2009 family income | 0.52 (0.42-0.62) | 0.16 | 0.09 | 0.57 | 0.07 | 0.56 (0.47-0.66) | 0.18 | 0.11 | 0.61 | 0.07 |
| *Model 9*  2010 family income | 0.48 (0.39-0.59) | 0.16 | 0.09 | 0.53 | 0.08 | 0.58 (0.48-0.71) | 0.18 | 0.11 | 0.64 | 0.06 |
| *Model 10*  2011 family income | 0.54 (0.44-0.65) | 0.16 | 0.09 | 0.59 | 0.07 | 0.53 (0.44-0.65) | 0.18 | 0.10 | 0.59 | 0.07 |
| Note: PP1and PP2 are the predicted probabilities at 0.5 standard deviations below and above their means, respectively. Each model controls for age in years, immigrant status, marital status and parental education. Person and replicate weights are applied to each model. | | | | | | | | | | |

| **Supplementary table 2. Year-stratified analysis of family income on the presence of a longstanding illness or health problem in 2012** | | | | | | | | | | |
| --- | --- | --- | --- | --- | --- | --- | --- | --- | --- | --- |
|  | ***Women*** | | | | | ***Men*** | | | | |
|  | *OR (95% CI)* | *PP1* | *PP2* | *RR* | *RD* | *OR (95% CI)* | *PP1* | *PP2* | *RR* | *RD* |
| *Model 1*  2002 family income | 0.84 (0.77-0.92) | 0.43 | 0.39 | 0.91 | 0.04 | 0.91 (0.83-1.00) | 0.39 | 0.37 | 0.95 | 0.02 |
| *Model 2*  2003 family income | 0.84 (0.76-0.92) | 0.43 | 0.39 | 0.90 | 0.04 | 0.90 (0.81-0.99) | 0.39 | 0.37 | 0.94 | 0.02 |
| *Model 3*  2004 family income | 0.82 (0.75-0.90) | 0.43 | 0.39 | 0.90 | 0.04 | 0.88 (0.79-0.97) | 0.40 | 0.37 | 0.93 | 0.03 |
| *Model 4*  2005 family income | 0.83 (0.76-0.90) | 0.43 | 0.38 | 0.90 | 0.04 | 0.87 (0.78-0.97) | 0.40 | 0.37 | 0.92 | 0.03 |
| *Model 5*  2006 family income | 0.80 (0.73-0.88) | 0.43 | 0.38 | 0.88 | 0.05 | 0.89 (0.81-0.98) | 0.39 | 0.37 | 0.93 | 0.03 |
| *Model 6*  2007 family income | 0.81 (0.74-0.89) | 0.43 | 0.38 | 0.89 | 0.05 | 0.89 (0.80-0.98) | 0.39 | 0.37 | 0.93 | 0.03 |
| *Model 7*  2008 family income | 0.83 (0.76-0.91) | 0.43 | 0.38 | 0.90 | 0.04 | 0.88 (0.80-0.96) | 0.40 | 0.37 | 0.93 | 0.03 |
| *Model 8*  2009 family income | 0.80 (0.73-0.87) | 0.43 | 0.38 | 0.88 | 0.05 | 0.87 (0.79-0.95) | 0.40 | 0.36 | 0.92 | 0.03 |
| *Model 9*  2010 family income | 0.78 (0.71-0.84) | 0.43 | 0.37 | 0.86 | 0.06 | 0.88 (0.80-0.96) | 0.39 | 0.37 | 0.93 | 0.03 |
| *Model 10*  2011 family income | 0.77 (0.71-0.84) | 0.43 | 0.37 | 0.86 | 0.06 | 0.86 (0.77-0.95) | 0.40 | 0.36 | 0.91 | 0.03 |
| Note: PP1and PP2 are the predicted probabilities at 0.5 standard deviations below and above their means, respectively. Each model controls for age in years, immigrant status, marital status and parental education. Person and replicate weights are applied to each model. | | | | | | | | | | |
